# Germline TP53 mutational spectrum in French Canadians with breast cancer

**DOI:** 10.1186/s12881-015-0169-y

**Published:** 2015-04-12

**Authors:** Suzanna L Arcand, Mohammed R Akbari, Anne-Marie Mes-Masson, Diane Provencher, William D Foulkes, Steven A Narod, Patricia N Tonin

**Affiliations:** Cancer Research Program, The Research Institute of the McGill University Health Centre, 1001 Decarie Boulevard, E02.6217, Montreal, Quebec H4A 3J1 Canada; Women’s College Research Institute, Women’s College Hospital, and University of Toronto, Toronto, Ontario Canada; Département de médecine, Université de Montréal, Montreal, Quebec Canada; Centre de recherche du Centre hospitalier de l’Université de Montréal et Institut du cancer de Montréal, Montreal, Quebec Canada; Division de gynécologie oncologique Université de Montréal, Montreal, Quebec Canada; Lady Davis Institute of the Jewish General Hospital, Montreal, Quebec Canada; Program in Cancer Genetics, Departments of Oncology, Human Genetics and Medicine, McGill University, Quebec, Canada

**Keywords:** TP53, Breast cancer, Hereditary breast cancer, French Canadian, Founders, BRCA1, BRCA2

## Abstract

**Background:**

Specific germline mutations in the hereditary breast-ovarian cancer susceptibility (HBC/HBOC) genes, BRCA1, BRCA2 and PALB2, have been shown to recur in French Canadians of Quebec, Canada, and this has been attributed to common ancestors. Germline TP53 mutation carriers are known to segregate in Li-Fraumeni syndrome families, which feature young age of onset breast cancer. We have reported rare TP53 mutation carriers in French Canadian HBC families, though none recurred possibly due to the limited number of cancer families investigated. Here we describe TP53 germline mutations found in French Canadian cancer families provided from hereditary cancer clinics; investigate 37 new BRCA1 and BRCA2 mutation-negative HBC/HBOC families for the TP53 mutations; and assess the frequency of TP53 mutations in a 1235 French Canadian breast cancer cases not selected for family history of cancer.

**Methods:**

TP53 mutation-positive pedigrees from French Canadian cancer families were provided from local hereditary cancer clinics. Bidirectional Sanger sequencing of all protein encoding exons of TP53 was performed using peripheral blood lymphocyte DNA from breast/ovarian cancer probands from 37 HBC/HBOC families of French Canadian descent. Targeted bidirectional Sanger sequencing assay of regions containing the identified TP53 mutations was performed on 1235 French Canadian breast cancer cases not selected for family history cancer.

**Results:**

Five new TP53 mutations were identified in six pedigrees from hereditary cancer clinics. No deleterious mutations were identified in cancer probands from 37 HBC/HBOC families. A targeted mutation screen of the 1235 breast cancer cases identified a c.844C>T [p.Arg282Trp] mutation carrier. This mutation was also found among the six mutation-positive cancer families provided by the local hereditary cancer clinics. The targeted screen also uncovered a new TP53 mutation, c.685T>C [p.Cys229Arg] that was found in two breast cancer cases. All TP53 mutation carriers were among the 656 women with breast cancer diagnosed less than 50 years of age.

**Conclusions:**

In all six new TP53 mutations were identified in French Canadians, where two each occurred in independently ascertained cases/families. Although all newly identified breast cancer mutation carriers reported a family history of cancer, none were consistent with features of Li-Fraumeni syndrome families.

## Background

Germline TP53 mutations have been reported in rare instances of hereditary breast cancer (HBC) and hereditary breast and ovarian cancer (HBOC) syndrome families found negative for mutations in the BRCA1 and BRCA2 breast and ovarian cancer susceptibility genes. TP53, BRCA1 and BRCA2 mutation carriers share in common a significant risk for premenopausal breast cancer with estimated lifetime risks of 80-90%, 60-85% and 40-85%, respectively [1]. The rarity of TP53 mutation carrier HBC/HBOC families reflects the estimated 0.025% frequency of mutation carriers in the population [1]. This is in stark contrast with the estimated 0.1% frequency of BRCA1/BRCA2 mutation carriers in the population and is consistent with the observation that a significant number of HBC/HBOC families (>40%) harbor mutations these genes [1]. Although other breast cancer risk alleles of moderate penetrance have recently been identified, only BRCA1 and BRCA2 are routinely evaluated in genetic counseling settings in order to identify carriers for purposes of assessing cancer risk and offering management strategies for cancer detection and prevention [2]. Rarely are TP53 mutations investigated in HBC/HBOC families due the estimated low frequency (<1%) of carriers in breast cancer cases and the observation that 70% of carriers are found in Li-Fraumeni Syndrome (LFS) families [1]. In addition to premenopausal breast cancer, LFS families feature a wide spectrum of cancer types with an excess of young age of onset soft tissue and bone sarcomas, brain tumors and adrenocortical carcinomas [3]. However, breast cancer cases in the context of LFS accounts for less than 0.1% of all such cancer cases [1]. Thus TP53 mutation-positive LFS families may not identify all possible mutation carrier breast cancer cases.

Identifying TP53 mutation carriers is important as evidence suggests that mutation carrier LFS patients have an abnormal response to low dose radiation and should avoid radiation therapy whenever possible in order to limit the risk of secondary radiation-induced malignancies [4]. This finding raises the question of mutation screening practices. Mutation screening is recommended in the context of LFS and similar syndromes (LFS-like syndrome (LFL)) with overlapping clinical features of core cancers but not necessarily features of HBC/HBOC [1,3,5]. For example, the American Cancer Society recommends magnetic resonance imaging (MRI) for breast cancer screening in mutation carrier women in LFS families [6].

Routine screening for germline TP53 mutations in breast cancer cases has not been advocated due to the low estimated frequency of carriers. The frequency of carriers in the population is currently unknown. Various estimates have been made based on the genetic analyses of LFS families, breast cancer families, and often from age-selected cases of breast cancer [1,5]. In a recent study of 28 women with diagnoses of breast cancer before age 30 years, TP53 mutation carriers were found in 33.3% of the cases where 7.7% did not meet criteria for LSF [7]. In the United Kingdom, it is estimated that 6% of women with breast cancer under age 31 years carry TP53 mutations, and based on this evidence, a prevalence of 1/10,000 to 1/25,000 live births harboring pathogenic mutations was estimated [8]. The significance of mutation carriers the young age of onset breast cancer has resulted in recommendations from the National Comprehensive Cancer Network® for clinical practice of genetic/familial high risk cases of breast and ovarian cancers to test (concurrent with or following genetic testing of BRCA1 and BRCA2) women with breast cancer diagnoses less than age 35 years of age [9].

We have reported germline TP53 mutations in 3.8% of 52 HBC/HBOC families of French Canadian descent found negative for BRCA1 and BRCA2 mutations [10]. Our initial study was prompted by the observation of a TP53 mutation-positive HBC French Canadian family that also exhibited LFS features [10]. However, none of the two subsequently identified mutation-positive families in our study, exhibited features of the core cancers found in LFS families [10]. The carrier frequency of TP53 mutations in the French Canadian population of Quebec is unknown. A targeted analysis of c.638G>A [p.Arg213Gln] and c.869G>A [p.Arg290His], mutations initially found in a TP53 mutation screen of HBC families, did not identify any additional carriers among the 381 French Canadian women with breast cancer before age 50 years that were not selected for a family history of cancer [10]. The French Canadian population is known for exhibiting strong founder effects as indicated by the recurrence of specific BRCA1 and BRCA2 germline mutations in HBC/HBOC families [11,12]. Since our report of TP53 mutations, a specific mutation in a moderately penetrant breast cancer susceptibility gene, PALB2 (c.2323C>T [p.Gln775Ter]), has been found to recur in French Canadian HBC families and breast cancer cases [13,14]. Although there is currently no evidence of recurring TP53 mutations in this founder population, our report remains the only study that has focused on TP53 mutation analysis of French Canadian women with breast cancer [10].

To further characterized the spectrum of TP53 germline mutations in French Canadians, we describe the mutations found in hereditary cancer clinics, investigate breast/ovarian cancer probands from 37 new HBC/HBOC families found negative for BRCA1 and BRCA2 germline mutations, and report on the frequency of mutations found in a targeted screen of 1235 French Canadian breast cancer cases for TP53 mutations found in hereditary cancer families. Five new TP53 mutations in French Canadian cancer families were identified among the six pedigrees provided by local hereditary cancer clinics. No new TP53 mutations were identified in the 37 probands from new HBC/HBOC families. However, a screen of genomic regions found mutated in French Canadian cancer families identified a breast cancer case harboring the c.844C>T [p.Arg282Trp] mutation. This mutation was also identified among one of the six families (LFL) provided by the hereditary cancer clinics. Two breast cancer cases each carrying a c.685T>C [p.Cys229Arg] mutation were also found. All mutation carriers from screening the breast cancer cases were among the 656 women diagnosed less than 50 years of age. The results from this study will inform the development of mutation-screening policies for women with breast cancer in the Quebec population.

## Methods

### TP53 mutation carrier pedigrees from hereditary cancer clinics

**Figure 1:**
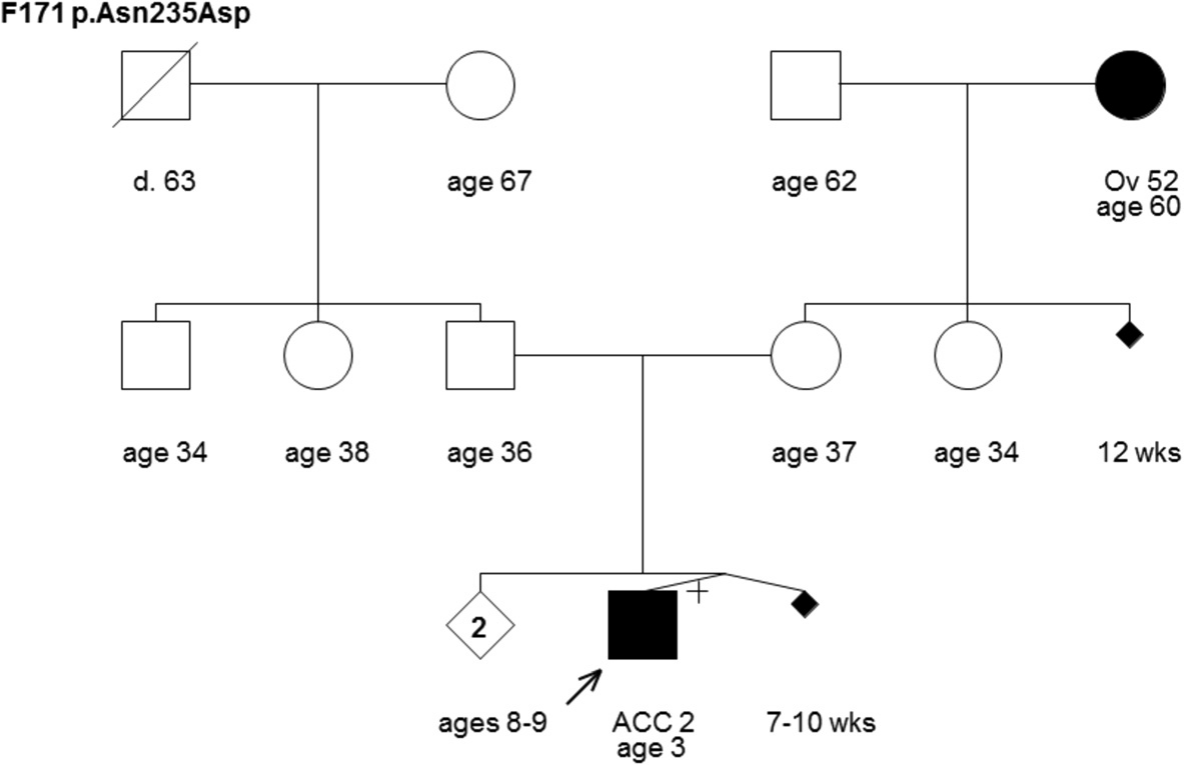
Pedigree of family F171 harboring the TP53 mutation c.703A>G [p.Asn235Asp]. Cancer type or site followed by age of diagnosis for adrenocortical carcinoma (ACC) and ovarian cancer (Ov). Age in years or weeks (wks) or age at death (d.) if known at time of ascertainment are shown.

**Figure 2:**
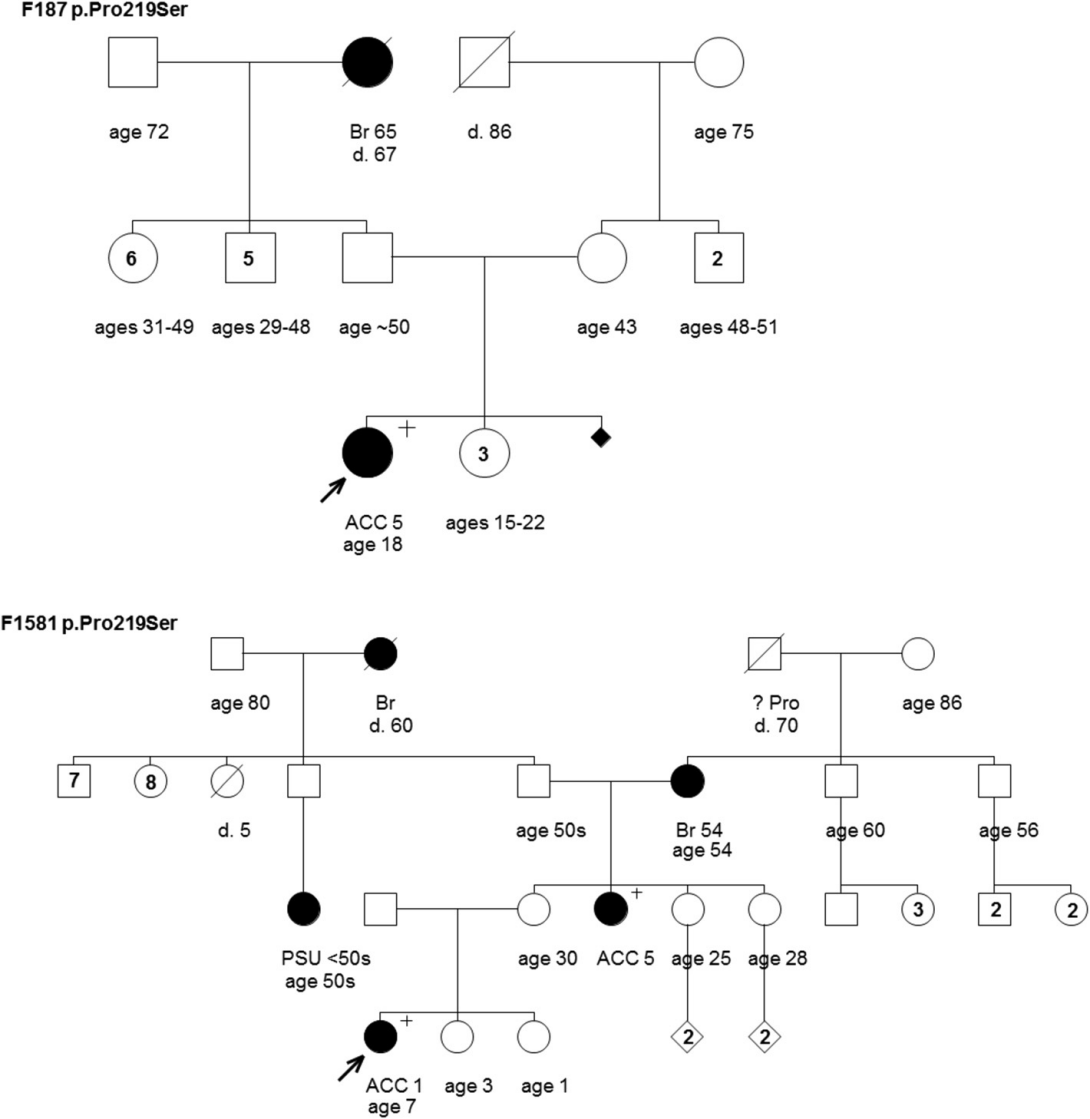
Pedigrees of families F187 and F1581 harboring the TP53 mutation c.655C>T [p.Pro219Ser]. Cancer type or site followed by age of diagnosis for adrenocortical carcinoma (ACC), breast cancer (Br), prostate cancer (Pro) and primary site unknown (PSU). Age in years or age at death (d.) if known at time of ascertainment are shown.

**Figure 3:**
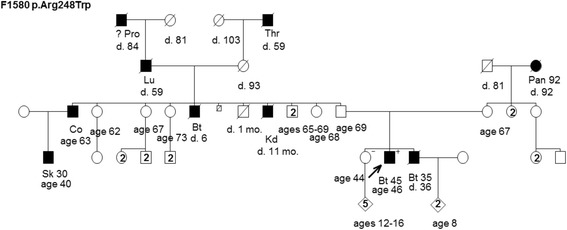
Pedigree of family F1580 harboring the TP53 mutation c.742C>T [p.Arg248Trp]. Cancer site followed by age of diagnosis for cancers of the brain (Bt), colon (Co), kidney (Kd), lung (Lu), pancreas (Pan), prostate (Pro), skin (Sk), and throat (Thr). Age in years or age at death (d.) if known at time of ascertainment are shown.

**Figure 4:**
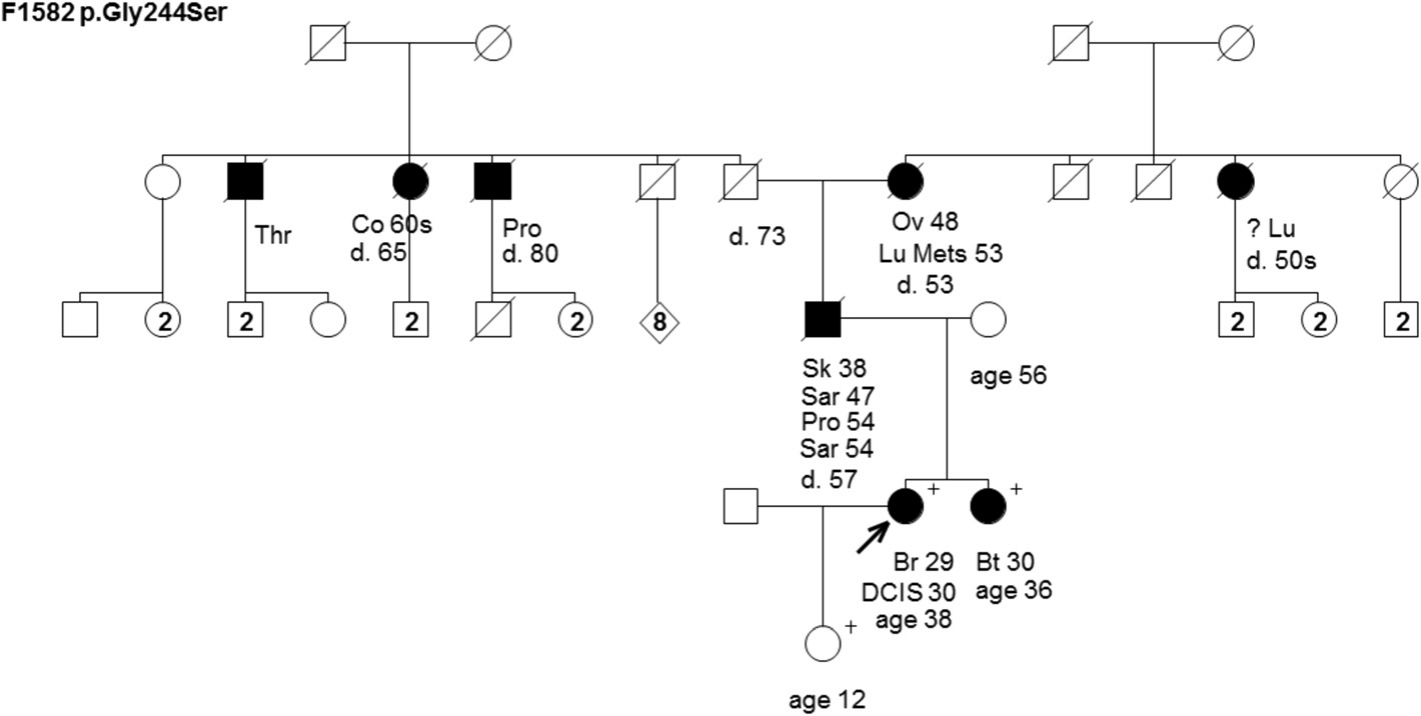
Pedigree of family F1582 harboring the TP53 mutation c.730G>A [p.Gly244Ser]. Cancer type or site followed by age of diagnosis for breast cancer (Br), brain tumor (Bt), colon cancer (Co), ductal carcinoma *in situ* (DCIS), lung cancer (Lu), lung metastasis (Lu Mets), ovarian cancer (Ov), prostate cancer (Pro), sarcoma (Sar), skin cancer (Sk), and throat cancer (Thr). Age in years or age at death (d.) if known at time of ascertainment are shown.

**Figure 5:**
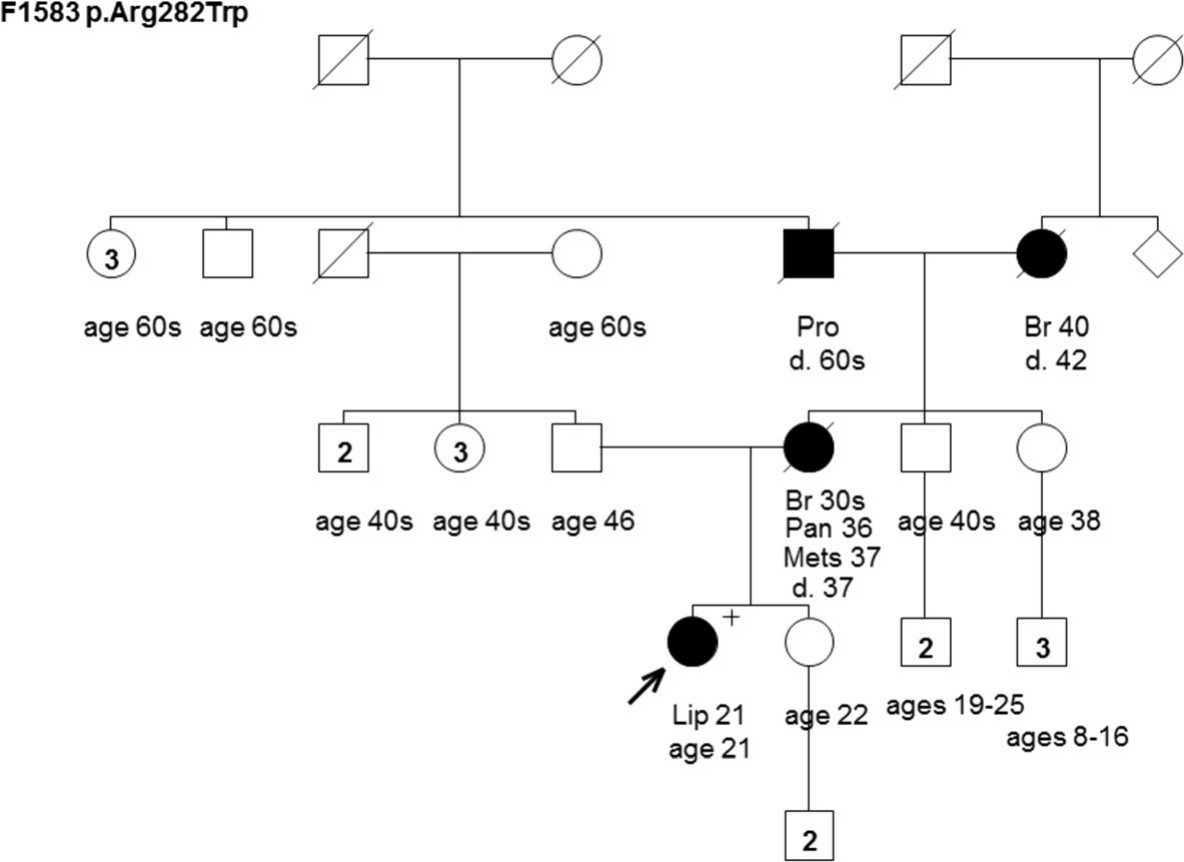
Pedigree of family F1583 harboring the TP53 mutation c.844C>T [p.Arg282Trp]. Cancer type or site followed by age of diagnosis for liposarcoma (Lip), breast cancer (Br), metastatic lesions (Mets), pancreas cancer (Pan), and prostate cancer (Pro). Age in years or age at death (d.) if known at time of ascertainment are shown.

Pedigrees from TP53 mutation-positive carriers of French Canadian descent from the Quebec population were requested from hereditary cancer clinics. From this request, six TP53 mutation carrier pedigrees (F171, F187, F1580, F1581, F1582 and F1583) were provided to the study by the hereditary cancer clinics affiliated with the McGill University Health Centre (MUHC), Jewish General Hospital and Centre hospitalier de l'Université de Montréal (CHUM) (Figures 1,2,3,4 and 5). All index cases carrying a TP53 mutation in the pedigrees self reported French Canadian (Quebec) ancestry. The pedigrees were provided along with the TP53 mutation results as indicated on the pedigrees, and not all cancer phenotypes indicated on the pedigrees (to our knowledge) were confirmed. The adrenocortical carcinomas (ACC) cases in pedigrees F171 (Figure 1) and F187 (Figure 2) were described in an earlier study of the role of germline TP53 mutations in childhood ACC but the pedigrees had not been previously reported [15]. The proband in Family F1581 had been tested due to prior knowledge of a TP53 mutation carrier in a family with childhood ACC (Figure 2). In the context of this study, this individual was found related to members of pedigree F187 (as described further in the Results section). The remaining three families (F1580, F1582, and F1583) that were provided to the study were tested for TP53 because they each had at least one LFS spectrum cancer.

Table 1 includes information for all TP53 mutation positive pedigrees that were provided by the hereditary cancer clinics as described above. Table 1 also includes information for three other pedigrees (F875, F1039 and F1444) that have been described previously in the context of the TP53 mutation screening study of HBC families from our group [10]. The recruitment criteria for these pedigrees have been described (see Study Subjects for familial cases below) [10]. As F875, F1039 and F1444 pedigrees were published previously [10] they are not reproduced in this report.

**Table 1:** **TP53 mutation spectrum in French Canadian cancer families and/or cases**

**TP53 mutation designation**	**Amino acid codon alteration**	**Location of mutation (exon)**	**Cancer and age of diagnosis (years) of mutation carrier index case**	**Family pedigree number**	**Family history of breast and/or ovarian cancer**	**Comments**	**Source of family or case**	**Reference**
c.638G>A	p.Arg213Gln	6	BiBr38	F1039	yes	Related to F1444	A	[8]
c.638G>A	p.Arg213Gln	6	Br41	F1444	yes	Related to F1039	A	[8]
c.655C>T	p.Pro219Ser	6	ACC5	F187	yes	Related to F1581	B	[13]
c.655C>T	p.Pro219Ser	6	ACC1	F1581	yes	Related to F187	B	This study
c.685T>C	p.Cys229Arg	7	Br35;Br38	F1602	yes		C	This study
c.685T>C	p.Cys229Arg	7	Br46	F1603	yes		C	This study
c.703A>G	p.Asn235Asp	7	ACC2	F171	yes		B	[13]
c.730G>A	p.Gly244Ser	7	Br29	F1582	yes		B	This study
c.742C>T	p.Arg248Trp	7	Bt45	F1580	no		B	This study
c.844C>T	p.Arg282Trp	8	Lip21	F1583	yes		B	This study
c.844C>T	p.Arg282Trp	8	Br30;Br38	F1604	no		C	This study
c.869G>A	p.Arg290His	8	Br44	F875	yes		A	[8]

Source of index cases from HBC families reported in a previous study of TP53 mutation (A) [8], new cancer families from hereditary cancer clinics (B) or from breast cancer cases not selected for family history of cancer [(C); Index cases diagnosed with adrenocortical carcinoma (ACC), bilateral breast cancer (BiBr), breast cancer (Br), brain tumor (Bt), or liposarcoma (Lip).

### Study subjects

One group of study subjects was comprised of cancer-affected index cases from 37 independently ascertained HBC/HBOC cancer families. The index cases were recruited through the hereditary cancer clinics affiliated with the MUHC and CHUM as part of research studies of BRCA1/BRCA2 mutations in families of French Canadian (Quebec) origin as described previously [10,16]. There were 25 HBC and 12 HBOC families defined as follows: in addition to the index case affected with breast cancer at less than 66 years of age (n = 33) or ovarian cancer case at any age (n = 4), the families had at least two other confirmed cases of invasive breast (less than 66 years of age) and/or epithelial ovarian cancer in the same familial branch. An index case from each family had been evaluated for BRCA1 and BRCA2 mutations by commercial DNA sequencing (Myriad Genetics, Myriad Genetics Laboratories, Salt Lake City, UT, USA) and was found negative or found to harbor a variant of unknown clinical significance [12,16]. Genomic DNA was provided by the Banque de tissus et de données of the Réseau de recherche sur le cancer of the Fonds de recherche du Québec - Santé.

Another group of study subjects was comprised of 1235 French Canadian women with invasive breast cancer that were not selected based on a family history of cancer. In this group were 656 women that were diagnosed before 50 years of age. They were recruited from a breast cancer out patient clinic affiliated with the CHUM and family history of cancer was collected as described previously [14,17]. Among these 1235 patients, there were 62 known carriers of BRCA1, BRCA2, PALB2 or CHEK2 mutations [14,17].

Informed consent was obtained from all study subjects and the study was performed in accordance with the guidelines established by the respective institutional ethical review boards.

### TP53 Mutation analyses

A total of 37 probands from HBC/HBOC families were investigated for TP53 germline mutations. The mutation analysis of the index cases from 36 HBC/HBOC cancer families was designed to detect variants in the protein coding exons 2 to 11, and adjacent splice sites of TP53 as described previously [18]. Briefly, PCR-based assays were followed by bidirectional sequencing using the 3730XL DNA Analyzer system (Life Technologies Inc., Burlington, Canada) at the McGill University and Genome Quebec Innovation Center (http://gqinnovationcenter.com). Commercial sequencing was performed for an index case from one of the HBOC families.

A targeted TP53 mutation analysis was performed for the 1235 breast cancer cases not selected for family history of cancer. This analysis was designed to detect the mutations that were found in French Canadian cancer families that were provided from the hereditary cancer clinics (see Table 1). As described above, mutation detection was based on bidirectional Sanger sequencing of PCR products derived from the amplification of exons 5 to 9.

Sequence chromatograms were compared with the NCBI reference sequence (RefSeq) reported in GenBank: NM_000546.4 and the genomic structures available from the February 2009 GRCh37/hg19 assembly of the human reference genome. Sequence variants were compared with allele frequencies reported in the Single Nucleotide Polymorphism (SNP) Database (www.ncbi.nlm.nih.gov/SNP), NHLBI Exome Sequencing Project (ESP): Exome Variant Server (EVS) Database (http://evs.gs.washington.edu/EVS/), and information available in the International Agency for Research on Cancer (IARC) TP53 Database version R17 November 2013 [19].

## Results

### Case review identifies new TP53 mutations in French Canadian cancer families

In a genetic analysis of TP53 we initially reported three mutation carrier HBC families of French Canadian descent (F875, F1039 and F1444), where F1039 and F1444 harbored the same c.638G>A [p.Arg213Gln] mutation and were found to be closely related following a case review (Table 1) [10]. To further characterize the spectrum of TP53 mutations in the French Canadian population of Quebec, we requested mutation positive pedigrees from hereditary cancer clinics based in Montreal, Quebec. Six TP53 mutation positive pedigrees (F171, F187, F1580, F1581, F1582 and F1583) where provided to the study along with carrier status and familial cancer phenotypes (Figures 1,2,3,4 and 5). None of these pedigrees carry the same mutations found in our previously reported HBC families (Table 1). All mutations are missense and predicted to affect the DNA binding domain of p53 based on the IARC TP53 Database.

Three of pedigrees provided by the hereditary cancer clinics had at least one pediatric ACC case (F171, F187 and F1581) (Figures 1 and 2). As mentioned above (see Methods), the ACC cases in families F171 and F187 were tested for TP53 in the context of a study evaluating germline TP53 mutation in this disease [15]. Interestingly, families F187 and F1581 both harbored the same mutation, c.655C>T [p.Pro219Ser] (Figure 2). A review of the pedigrees and case histories suggested that these families are related through the ACC case diagnosed at five years of age. The mutation was originally identified in this ACC case and reported in a 1994 study, along with another ACC case described in the unrelated family F171 (Figure 1), which harbored a different mutation (c.703A>G [p.Asn235Asp]) ) (Table 1) [15]. The pedigrees shown depict a cancer family history reported at the time the study was conducted. Notable is that each of these families reported a second-degree relative with breast or ovarian cancer. Additional cancer cases have since been reported for the ACC case linked to family F187 (Figure 2). Family F1581 (Figure 2) is notable for the further development of other ACC and breast cancer cases since it was initially presented (as F187).

The remaining families that were provided from the hereditary cancer clinics (F1580, F1582 and F1583 (Figures 3,4 and 5)) had cancer cases consistent with LFS syndrome, such as pediatric or young age of onset brain tumors and sarcomas, and each family harbored a unique TP53 mutation (Table 1). Although the confirmation of cancer cases was not provided to the study, we were informed that the presence of LFS spectrum cancer rationalized genetic testing of TP53 in the hereditary cancer clinics.

Five of the six families, the exception being F1580 (Figure 3) provided by the hereditary cancer clinics, each had reported a case of breast and/or ovarian cancer (Table 1). Family F1580 (Figure 3) is striking for the number of reported cases of males with cancer. Gender bias in TP53 mutation carriers has been reported in independent studies, where penetrance appears to be higher in females than males perhaps due to the high penetrance for female breast cancer [20,21]. However, the mutation carrier status of all the individuals in carrier families and the familial origin of the TP53 mutation are unknown.

In all there were seven different TP53 mutations associated with French Canadian (Quebec) cancer families at the initiation of the present study.

### No new French Canadian mutation identified in screen of HBC/HBOC families

A mutation analysis of index cases from 37 HBC/HBOC families identified 14 sequence variants (Table 2). Eleven of these variants occurred within intronic sequences and two resulted in synonymous amino acid substitutions that are all classified as polymorphisms based on information from the IARC TP53, dbSNP and EVS Databases. The c.215G>C [p.Arg72Pro] polymorphism was found in heterozygous/homozygous state in 41.7% (n = 15) of cases. This polymorphism has been found to be of functional significance as the proline amino acid is less efficient than arginine at codon 72 at inducing apoptosis and this has been attributed to weaker binding and ubiquitination by MDM2 of the proline containing isoform [22,23]. Although the allele frequencies of these variants in the French Canadian population are not known, our results are comparable to a previous analysis of 52 probands from HBC/HBOC families analyzed for TP53 mutations from our group [10] and to those reported for European (Caucasian) populations as reported in the dbSNP and/or EVS Databases (Table 2). Thus no new deleterious mutations were identified in cancer cases representative of 37 French Canadian HBC/HBOC families found negative for deleterious BRCA1/ BRCA2 mutations.

**Table 2:** **Characterization of TP53 variants identified in index cases of French Canadian HBC/HBOC families**

**Genotype alteration**	**Amino acid codon alteration**	**Genetic location of variant**	**Variant genotype designation**	**Distribution of variant genotypes identified in French Canadian index cases** ^**1**^			**Variant allele frequencies identified in French Canadian index cases (%)**				**General population of variant allele frequencies** ^**2**^ **(%)**		**Source of general population variant allele frequencies** ^**2**^
				**AA**	**AB**	**BB**	**A**	**A%**	**B**	**B%**	**A%**	**B%**	
c.1-140G>A		intron 1	rs8079544	35	1	0	71	98.6	1	1.4	94.6	5.5	A
c.74+38C>G		intron 2	rs1642785	3	12	21	18	25.0	54	75.0	26.0	74.0	B
c.96+41_96+56del16		intron 3	rs17878362	24	11	1	59	81.9	13	18.1	79.5	20.5	B
c.97-29C>A		intron 3	rs17883323	35	1	0	71	98.6	1	1.4	94.8	5.2	B
c.108G>A	p.Pro36Pro	exon 4	rs1800370	34	2	0	70	97.2	2	2.8	98.5	1.5	B
c.215G>C	p.Arg72Pro	exon 4	rs1042522	21	12	3	54	75.0	18	25.0	78.3	21.7	A
c.376-91G>A		intron 4	rs2909430	0	7	17	7	14.6	41	85.4	9.9	90.1	A
c.639A>G	p.Arg213Arg	exon 6	rs1800372	33	3	0	69	95.8	3	4.2	98.1	1.9	B
c.672+62A>G		intron 6	rs1625895	1	9	26	11	15.3	61	84.7	10.3	89.7	A
c.673-36G>C		intron 6	rs17880604	35	1	0	71	98.6	1	1.4	98.4	1.6	B
c.782+72C>T		intron 7	rs12947788	30	6	0	66	91.7	6	8.3	-	-	-
c.782+92T>G		intron 7	rs12951053	30	6	0	66	91.7	6	8.3	92.4	7.6	A
c.993+12T>C		intron 9	rs1800899	35	1	0	71	98.6	1	1.4	98.5	1.5	B
c.1100+30A>T		intron 10	rs17880847	35	1	0	71	98.6	1	1.4	98.5	1.5	B

^1^Genotypes were not available (not included) for the index case screened in the hereditary cancer clinic; ^2^Population based allele frequencies from Hap Map CEU (A) (http://hapmap.ncbi.nlm.nih.gov/) or Exome Variant Server - European Americans (B) (http://evs.gs.washington.edu/EVS/); Population frequency was not known for rs12947788.

### Targeted mutation screen identifies TP53 mutation-positive breast cancer cases

**Figure 6:**
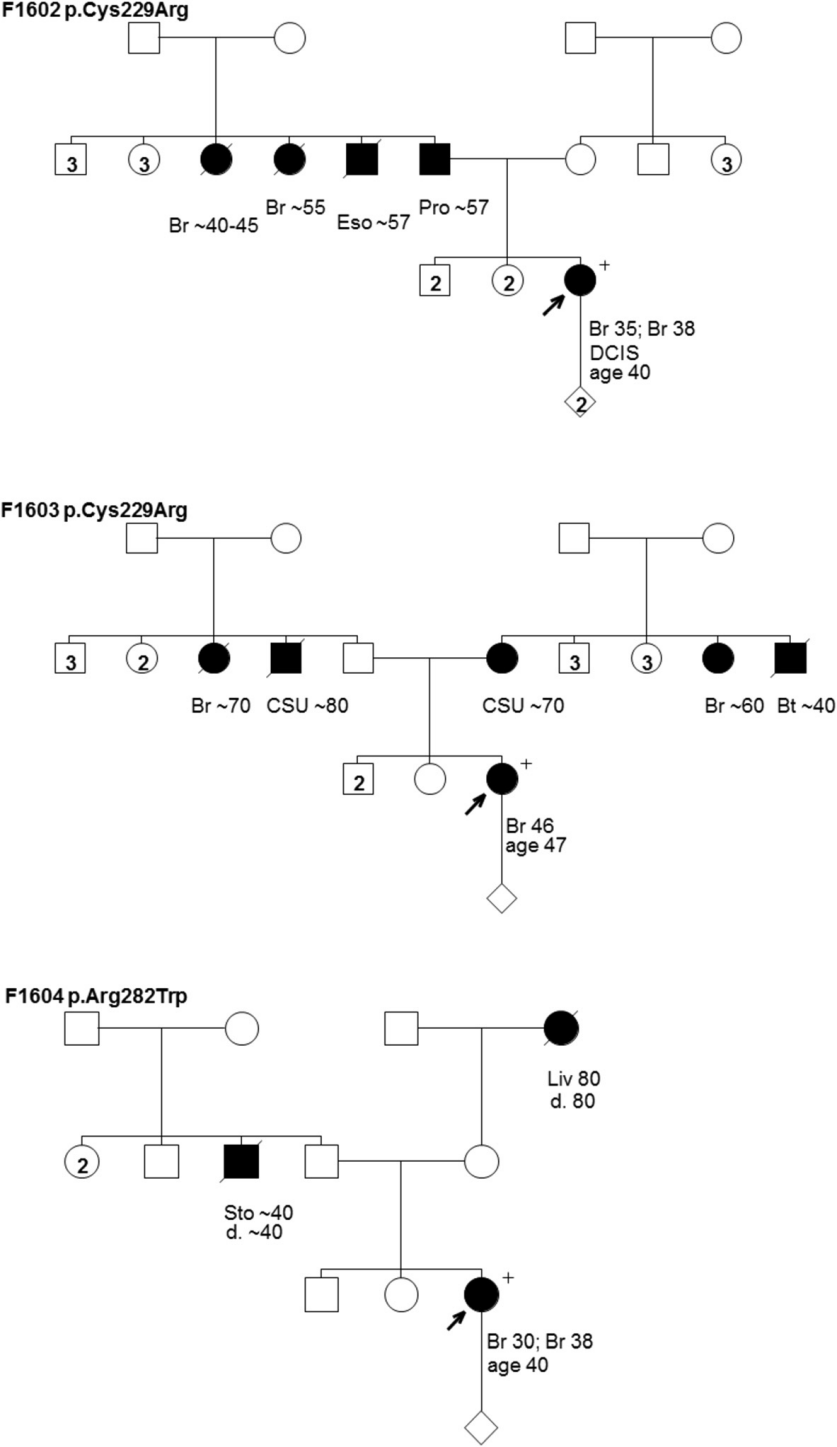
Pedigrees from breast cancer case reports of families F1602 and F1603 harboring the TP53 mutation c.685T>C [p.Cys229Arg] and family F1604 harboring the TP53 mutation c.844C>T [p.Arg282Trp. Cancer type or site followed by age of diagnosis for breast cancer (Br), brain tumor (Bt), cancer site unknown (CSU), ductal carcinoma *in situ* (DCIS), esophageal cancer (Eso), liver cancer (Liv), prostate cancer (Pro), and stomach cancer (Sto). Age in years or age at death (d.) if known at time of ascertainment are shown.

Three mutation carriers among the 1235 women with breast cancer not selected for family history of cancer were identified in a targeted TP53 mutation screen for regions containing mutations found in French Canadians (Table 1). All carriers were among the 656 women who were diagnosed under 50 years of age. Family histories of cancer were obtained at interview and this information is shown in Figure 6.

The c.844C>T [p.Arg282Trp] mutation is predicted to affect the DNA binding domain of p53 based on the IARC TP53 database (Table 1). The carrier of this mutation had breast cancer at ages 30 and 38, and reported an unremarkable family history of cancer: a paternal uncle with stomach cancer in his 40’s and a maternal grandmother with liver cancer at age 80 (Figure 6). Interestingly, the same mutation was found in one of six pedigrees (F1583) provided by the hereditary cancer clinics (Figure 5). Although it is not known if the carriers are related, their personal and family histories of cancer are not recognizable in a comparison of their pedigrees.

The same c.685T>C [p.Cys229Arg] mutation was found in two women with breast cancer not selected for cancer family history (Table 1). This mutation is predicted to affect the DNA binding domain of p53 based on the IARC TP53 database. The familial relationship of these carriers is unknown and their reported family histories of cancer are shown in pedigrees F1602 and F1603 (Figure 6). The carrier from family F1602 had breast cancer at ages 35 and 38, which included a ductal carcinoma *in situ*. This carrier reported a father with prostate cancer estimated to have occurred at ages 57–58, two paternal aunts with breast cancer between 40–55 years of age, and a paternal uncle with esophageal cancer at age 57 (Figure 6). The carrier from family F1603 was diagnosed with breast cancer at age 46. This carrier reported both a paternal and a maternal aunt with breast cancer between 60–70 years of age, a paternal uncle with an unspecified cancer at age 80, and a maternal uncle with brain cancer at age 40. Thus both of these independently ascertained mutation carriers had a family history of breast cancer.

## Discussion

Although the carrier frequency of TP53 mutations in the French Canadian population of Quebec is currently unknown, a picture is emerging of the contribution of this gene to hereditary breast cancer. In previous studies we reported that 3.8% (2/52) of HBC/HBOC families negative for BRCA1 and BRCA2 mutations harbored TP53 mutations [10]. None of the 37 families evaluated in the present study were found to carry variants predicted to have deleterious effect on p53 function. As the ascertainment criteria of HBC and HBOC families is the same across all of our studies in this population, our revised estimate of the carrier frequency in HBC/HBOC with at least three cases of breast cancer under 66 years or age and/or ovarian cancer found negative for BRCA1 and BRCA2 is approximately 2.2% (2 of 89). Although our ascertainment criteria differ from independent reports, this frequency is consistent with the low carrier frequency in HBC/HBOC families found negative for germline BRCA1 and BRCA2 mutations [1].

The TP53 mutations c.685T>C [p.Cys229Arg] and c.844C>T [p.Arg282Trp] identified in a screen of women with breast cancer not selected for family history of cancer are both predicted to effect the DNA binding domain of p53. The clinical significance c.685T>C [p.Cys229Arg] mutation is unknown as various bioinformatic tools render neutral to partial functionality of the encoded protein as reported in the IARC TP53 mutation database. However, this mutation has been reported to occur in somatic cells in the IARC TP53 mutation database and was also reported as a germline mutation in a LFS family [24]. In contrast, c.844C>T [p.Arg282Trp] is predicted to have a deleterious effect on protein function and has been reported as a common somatic and germline mutation in the IARC TP53 mutation database. Interestingly, this mutation was found in family F1583, a carrier family provided to the study by hereditary cancer clinics (see Table 1, Figure 5). The relationship between the carriers of the same mutation is unknown. The mutation carrier in F1583 had a liposarcoma at age 21 and her mother had breast cancer in her 30’s and pancreatic carcinoma at age 36 (Figure 5). However, the mutation carrier status of her mother or other cancer-affected individuals in the family is unknown. It is tempting to speculate that when taken together the mutations identified in breast cancer cases not selected for family history of cancer are likely pathogenic.

Our findings suggest that less than 0.5% of French Canadian (Quebec) women with breast cancer carry a germline TP53 mutation. Our study was limited to an evaluation of exons five to nine. However a mutation screen of these exons covers the regions that encode the DNA binding domain of p53, which we estimate accounts for 74% of germline TP53 mutations according to IARC TP53 Mutation Database. We have not included in our estimate, carriers of the c.215G>C [p.Arg72Pro], a variant found to be of biological significance [22,23]. However, its role as a modifier of cancer risk is controversial, as large case control studies by the Breast Cancer Association Consortium found no significant association with breast cancer risk [25] or breast cancer risk in BRCA1/BRCA2 mutation carriers [26]. Our carrier frequency is in keeping with independent mutation screens of breast cancer cases not selected for family history of cancer or ethnicity [1]. The observation that all mutation carriers were among the women diagnosed under age 50 years is also in keeping with independent reports and is a reflection of the high penetrance of mutated TP53 alleles, which is estimated to confer an 18–60 fold increased risk of breast cancer [1-3].

The estimated carrier frequency for women diagnosed with breast cancer under 50 years of age, at 0.5%, is lower than that estimated for BRCA1/BRCA2 carriers in the same population. A study of 564 French Canadian women with breast cancer under 50 years of age reported a 4.8% carrier frequency in a mutation screen for eight BRCA1/BRCA2 mutations found to recur in French Canadian HBC/HBOC cancer families [14]. Within this cohort 0.7% and 1.1% were found to harbor either the PALB2:p.Gln775Ter or CHEK2:c.1100delC recurrent French Canadian mutations, respectively [14]. A more recent study from the same group, found that 5.3% of French Canadian women with invasive breast cancer had one of six recurrent BRCA1/BRCA2 mutations [17]. The mutation frequency may be higher, given that the screen did not include an analysis for BRCA2:p.Glu3002Lys, a mutation that has been recently reclassified as pathogenic and found to recur at a low frequency in French Canadians [27,28]. Thus relative to BRCA1/BRCA2, germline TP53 mutations are infrequent in French Canadian women with breast cancer, and may be similar (if not lower than) the mutation frequencies observed for specific PALB2 and CHEK2 mutations observed for this population.

The identification of carriers with the same c.685T>C [p.Cys229Arg] or c.844C>T [p.Arg282Trp] mutation in TP53 is not surprising given the founder effects observed in the French Canadian population [29]. Reports of recurrent TP53 mutations due to common founders have been rare [5]. For example, the recurrence of p.Arg337His variant appears to be the cause of the high incidence of childhood ACC in Southeastern Brazil [30,31]. However, the familial relationship of the carriers of identical TP53 mutations in our study is not known. The low frequency of carriers and broad spectrum of mutations identified does not warrant a targeted screen for these mutations at the present time, as has been recommended for recurrent mutations in BRCA1 and BRCA2 [12,14,17]. Also, it is possible that the carriers are closely related as was discovered in our previous study of independently ascertained families F1444 and F1039 [10] and in a case review of families F1581 and F187 in the present study.

Our results raise the question of routine TP53 mutation testing in women with breast cancer. None of the carrier women with breast cancer from the cohort not selected for family history of cancer, reported a family history of cancer that would have warranted an investigation of TP53 in clinical settings as has been recommended for LFS (Figure 6). The identification of TP53 mutation carriers would identify those women who are predicted to have an abnormal response to low dose radiation [4]. Moreover, it may identify family members at risk for breast cancer and other malignancies associated with LFS [5]. Counseling issues are complex with TP53 mutation carriers due to the spectrum of cancer type involved, particularly where there are few options for cancer surveillance or prevention [2]. In Canada, all genetic testing for TP53 (and BRCA1 and BRCA2) can only be ordered by cancer genetics professionals associated with a medical genetics clinic, which are managed by provincial health care jurisdictions. Currently there are no official testing guidelines in the province of Quebec and no routine testing of women with breast cancer. Women are referred to genetic counseling units for further assessment prior to genetic testing. It may be difficult to advocate genetic testing of breast cancers regardless of family history of disease in the province of Quebec, especially given the low frequency of mutation carriers. However, the risk for female breast cancer risk is high in mutation carriers and nonionizing cancer surveillance methods are available for carriers [2]. Recently recommendations by the National Comprehensive Cancer Network® have been made to revise practice guidelines, which recommend TP53 mutation testing for women with breast cancer diagnoses under of the age of 35 years [9]. However these guidelines when applied to women with breast cancer in our study would have identified only three of the seven mutation carriers (see Table 1). As mutation analysis becomes more cost effective through the use of multiplexed mutation screens or whole exome sequencing, our findings would support the screening of TP53 as part of the panel of breast cancer susceptibility genes investigated in high risk women and women with breast cancer.

## Conclusions

New germline TP53 mutations were identified in French Canadians with cancer, a population that exhibits a unique genetic demography due to common founders. Mutation carriers included women diagnosed with breast cancer before 50 years of age. None of these women had a family history of cancer consistent with LFS. Our findings further define the spectrum and frequency of germline TP53 mutations in women with breast cancer not selected for family history of cancer and could further inform the development of mutation screening policies for French Canadians in Quebec.
